# Incorporating the voice of young people in mental health research: reflections from three lived experience advisory panels in Latin America

**DOI:** 10.1186/s40900-025-00703-5

**Published:** 2025-04-17

**Authors:** Liliana Hidalgo-Padilla, Daniela Ramirez-Meneses, Karen Ariza-Salazar, Santiago Cesar Lucchetti, Natividad Olivar, Diliniya Stanislaus Sureshkumar, Catherine Fung, Luis Ignacio Brusco, Carlos Gómez-Restrepo, José Miguel Uribe-Restrepo, Stefan Priebe, Francisco Diez-Canseco

**Affiliations:** 1 CRONICAS Center of Excellence in Chronic Diseases, Universidad Peruana Cayetano Heredia, Lima, Peru; 2 Department of Clinical Epidemiology and Biostatistics, Pontificia Universidad Javeriana, Bogotá, Colombia; 3 Department of Psychiatry and Mental Health, Faculty of Medicine, University of Buenos Aires, Buenos Aires, Argentina; 4 Unit for Social and Community Psychiatry, Wolfson Institute of Population Health, Queen Mary University of London, London, UK; 5 Hospital Universitario San Ignacio, Bogotá, Colombia; 6 Department of Psychiatry and Mental Health, Pontificia Universidad Javeriana, Bogotá, Colombia; 7 Centre for Psychosocial Medicine, University of Hamburg, Hamburg, Germany

**Keywords:** Lived experience, Participation, Public, Patient participation, Mental health, Americas

## Abstract

**Background:**

Involving people with lived experience in research has been increasingly recognised as a priority. This article details the development and implementation of three Lived Experience Advisory Panels (LEAP) and reports their evaluation of the experience. The LEAPs involved young people from Latin America with experiences of emotional distress, aimed at advising a youth mental health research programme.

**Methods:**

Online meetings were conducted within each LEAP in Bogotá, Lima and Buenos Aires to gather feedback at different stages of the programme. Additional activities included a Joint LEAP meeting and a focus group to explore the members’ experiences of LEAP participation.

**Results:**

During the LEAP meetings, 25 members (Bogotá *n* = 9, Lima *n* = 10, Buenos Aires *n* = 6) provided feedback on study data collection tools and materials, recruitment and dissemination strategies, and discussed preliminary results, which were significant in shaping study materials and processes. They valued the comfort and connection with researchers and peers during discussions about mental health topics, as well as the opportunity to inform a large-scale project with their lived experiences. However, challenges in the recruitment and engagement led to fluctuating participation, while socioeconomic and cultural barriers may have influenced access.

**Conclusions:**

The implementation of the LEAPs is an example of involving young Latin Americans with experiences of emotional distress in research. Future efforts should focus on overcoming structural limitations and fostering equitable collaboration, emphasising the need for youth perspectives in mental health research, particularly in low- and middle-income countries.

**Supplementary Information:**

The online version contains supplementary material available at 10.1186/s40900-025-00703-5.

## Abstract (plain language summary)

Involving people with personal experiences of mental health issues in research is becoming more important. They are considered “experts” based on their knowledge from living with a health condition and dealing with the healthcare system to receive treatment. However, much of this research occurs in wealthier countries, where public participation is more common. This paper describes and examines the development and implementation of three Lived Experience Advisory Panels (LEAPs) with young Latin Americans who have faced emotional distress in a mental health research programme, and reports their evaluation of the experience.

The study highlights the opportunity to engage young people in mental health research in Latin America. LEAP members helped improve tools for collecting data, gave feedback on how to recruit participants and share findings, and participated in pilot studies. Despite successfully establishing the LEAPs, we encountered challenges, such as needing to re-think our recruitment strategies and considering socioeconomic and cultural factors that might affect participation. The experience was influenced by power dynamics that we need to be aware of throughout the process. Still, LEAP members appreciated the safe space to talk and connect with others who shared similar experiences, and they valued the opportunity to contribute to a large-scale project. Looking back on the experience, it is clear that our impact was limited, highlighting the need for researchers to empower individuals with lived experience, particularly young people, to ensure they have a significant role in future research.

**Supplementary Information:**

The online version contains supplementary material available at 10.1186/s40900-025-00703-5.

## Background

In recent years, the participation of people with lived experience in the design and development of health research has become essential to meet target populations’ needs [1–3]. The term “people with lived experience” refers to people experiencing health problems, who may or may not be users of health services, and their carers [4]. They are considered “experts” because of the experience and knowledge they have acquired from living with a health condition and navigating the health system to receive treatment [5, 6].

Lived experience is not a new concept in mental health research [7]. In the early 2000s, Mad Studies were coined as an interdisciplinary field that critically examines how social and medical systems contribute to the onset of mental illness [8]. It emerges from a longer tradition of theory and activism focused on the experiences of people who identify themselves as ‘mad’, and seeks to create space in academia for the perspectives of people whose views have been systematically discarded [9, 10].

Nowadays, lived experience can be embedded in mental health research through various collaborative approaches. These range from lived experience advisory panels that guide research processes through collaboration with researchers [11] to more engaging approaches such as co-creation (where stakeholders help identify problems, develop solutions, and oversee implementation and evaluation); co-design (where stakeholders contribute to designing solutions for a defined issue); and co-production (where stakeholders collaborate in implementing agreed-upon solutions) [12]. People with lived experience can be involved at different levels, from providing input during specific research phases to actively shaping the entire research process, including design, data collection, analysis, and interpretation [13].

Lived Experience Advisory Panels (LEAPs) are a widely used approach in research. Typically, LEAP meetings are led by a chairperson who ensures the sessions are run effectively and all members have the opportunity to provide their input. These responsibilities might be assigned to a LEAP member or a research staff member [14]. There is evidence for LEAPs in mental health research. For example, a Canadian study formed a LEAP with people with depression who participated in formulating research questions, designing study materials and analysing data [2]. Another study in the United Kingdom (UK), carried out with a similar population, reported that the involvement of its LEAP increased progressively, going from solely consulting to co-production of some aspects of the study, such as developing assessment tools, training researchers about interviewing services users and disseminating findings [15]. Overall, the involvement of people with lived experience makes it possible to improve the quality of research planning, results, and dissemination [16].

It has been observed that young people are particularly less involved in collaborative initiatives to improve public policy or health services [17]. Their poor involvement in research could be due to being considered a vulnerable population, lacking training in research, or the legal regulations limiting their participation in such activities [18–20]. However, some studies do actively include them [21, 22], which is crucial to ensure future research is responsive to their particular needs [23]. For instance, a charity in England has a LEAP specifically comprising young people aged 13–28 years, who are involved in developing and advising mental health research proposals, ensuring these are appropriate, effective and relevant for the research target population [24].

Involvement in research can also positively impact people with lived experience. For example, interviews conducted with a LEAP from a mental health institute in Australia indicate that being part of this group allowed for listening to different perspectives (i.e. young people, carers, mothers, among others) and provided a safe and judgement-free space to discuss mental health outside of a treatment context [16]. Likewise, patients and carers from a comprehensive network for health research across England providing logistical support to research studies, the NIHR Clinical Research Network [25], commented on the social benefits of being part of these groups, as they developed new friendships and received support from their peers [26].

Generally, studies engaging people with lived experience are more prevalent in countries where user participation in health research is frequent [27]. Replicating this in Latin America would be valuable, as the level of involvement of young people in research, as well as the overall understanding of mental health problems in low- and middle-income countries (LMICs), differs from the high-income ones [28]. Additionally, the region shares a common language and faces similar social challenges, such as poverty and healthcare access disparities [29–31]. These factors are essential when shaping discussions in mental health research. However, we have not yet identified studies that report this type of experience in the region.

The aim of this article is to describe and examine the development and implementation of three LEAPs with young people with lived experience of emotional distress, and report their evaluation of the experience. The LEAPs were formed as part of the research programme ‘Building resilience and resources to reduce depression and anxiety in young people from urban areas in Latin America (OLA)’, hereinafter ‘OLA Programme’. This 5-year programme aimed to identify the resources and activities that help young people living in low-resource urban settings in Latin American cities (Buenos Aires, Argentina; Bogotá, Colombia; and Lima, Peru) to prevent or recover from depression and/or anxiety [32, 58]. The structure and activities conducted as part of the OLA Programme are described in Supplementary Material 1.

## Methods

### Purpose of the LEAPs

The research team identified the importance of incorporating young people’s voices in an advisory capacity. The aim of the LEAPs was to gather feedback from young individuals with current or past experiences of emotional distress (depression and/or anxiety) to inform study decisions. This included validating data collection tools and materials, suggesting implementation strategies, commenting on findings, and advising on dissemination. This article describes the experience across three stages: (a) development, (b) implementation, and (c) feedback from LEAP members.

### Development

At the beginning of the OLA Programme, all research teams met with the UK-based project manager who described their experience of developing LEAPs for research projects in the UK. Teams could consult this manager for guidance throughout the process of establishing or implementing their LEAPs.

Three LEAPs were established, one in each study city (Bogotá, Buenos Aires and Lima). To ensure relevant feedback on the study, recruitment targeted individuals mirroring the study’s target population [32, 58]. Inclusion criteria were: (a) aged 15 to 24 years; (b) residing in resource-constrained neighbourhoods of Bogotá, Buenos Aires or Lima; and (c) self-reporting lived experience of emotional distress.

The local research teams used snowball recruitment [33] by contacting educational institutions, arts organisations, community groups, mental health centres, and acquaintances of existing LEAP members. The LEAPs were advertised as opportunities for young people to support a regional mental health study, gain insights into mental health in Latin America, and develop social and organisational skills.

### Implementation

Local research teams aimed to organise LEAP meetings every six months over 5 years, each lasting approximately 90 min. Due to the COVID-19 pandemic, meetings were planned to take place via Zoom or Microsoft Teams. To ensure accessibility, meetings were compatible with laptops and mobile phones. Participants with limited internet access received mobile data to join the meetings. Additionally, members were encouraged to choose a quiet, comfortable location to minimise distractions and provide privacy. Once the social distancing measures were lifted, research teams explored the possibility of in-person meetings.

Meeting topics were agreed beforehand by the research teams based on the ongoing programme activities at the time, but there was flexibility to add topics relevant to each city. Members were never required to share personal experiences of distress; however, research teams were prepared to address such situations if needed.

Although meetings were conducted independently in each city, a joint meeting with the three LEAP groups was held to promote interaction between members and allow them to reflect on their countries’ current mental health situation.

#### Data sources

Demographic data (age, sex, and neighbourhood), meeting details (agenda, attendance, notes) and records of materials developed by LEAP members were recorded. Two researchers (LH, DR) categorised the LEAPs’ contributions to specific study activities. The notes from the joint LEAP meeting were also reviewed to summarise key themes.

### Young people’s feedback on the LEAPs

#### Data sources

A focus group to gather members’ opinions and recommendations based on their experience with the LEAP was conducted. Members who had attended at least three meetings were invited to participate, ensuring balance by country and gender. A topic guide was designed by the research teams to explore motivations for joining, perceptions of meetings and group dynamics, overall evaluation and recommendations. This was later revised by an external Peruvian researcher with extensive qualitative expertise, who also led the focus group to facilitate open feedback. The focus group was audio-recorded and transcribed verbatim.

#### Data analysis

The research team performed an inductive content analysis [34]. One early-career researcher (DR) with experience in qualitative coding developed a matrix in Excel to organise the collected information into categories. These were iteratively reviewed with a second researcher (LH) to ensure consistency until a final consensus was reached to structure the findings. Three categories emerged: (a) facilitators for LEAP engagement, (b) challenges, and (c) recommendations.

### Ethical considerations

The LEAP activities were approved by Institutional Ethics Committees at Queen Mary, University of London (QMERC2020/02), the Faculty of Medicine of the Pontificia Universidad Javeriana (FM-CIE-1138-20), Universidad de Buenos Aires, and Universidad Peruana Cayetano Heredia (E021-03-20). Adult LEAP members provided informed consent, while minors (under 18 years of age) provided assent with parental or guardian informed consent. Participation was voluntary, and members could withdraw at any point. Research teams covered transportation or mobile data costs whenever needed to reduce access barriers. After each meeting, including the focus group, members received a reimbursement equivalent to 10 USD for their time.

## Results

### Development

This was the first time the Latin American research teams implemented a LEAP. The LEAPs began their activities in the three cities in October 2020 and concluded in March 2025, at the end of the OLA Programme. Initial recruitment resulted in 6 members in Buenos Aires, 8 in Lima, and 11 in Bogotá. However, in Buenos Aires and Lima, some members did not attend the second meeting (February-March 2021), and none confirmed their attendance for the third (November 2021). Therefore, a second recruitment wave was conducted in July 2022, leading to 7 and 6 new members in Buenos Aires and Lima, respectively. Recruitment remained open throughout the study, allowing additional young people to join over time. Apart from the initial withdrawals in Buenos Aires and Lima, 6 additional members left the LEAP due to conflicting activities or inability to contact them.

By September 2024, there were 9 members in Bogotá, 7 in Buenos Aires, and 10 in Lima, with an average age of 22.1 years (SD = 2.8). Most members were women (65%), and all resided in low-resource neighbourhoods.

### *Implementation*

#### Meetings and attendance

Between October 2020 and September 2024, 24 LEAP meetings were conducted (9 in Bogotá and Lima, and 6 in Buenos Aires), which averaged 90 min and were scheduled on weekends to accommodate members’ study and work commitments. Initially, the meetings were planned every six months, but each team adjusted the frequency based on availability and study requirements. Two early-career researchers led the meetings, with the principal investigator in Colombia occasionally participating.

At the beginning, all meetings were online due to the restrictions arising from the COVID-19 pandemic, but this was maintained because members expressed it was easier to join online to avoid travel costs and time constraints. Members were encouraged to keep their cameras on during meetings, which was successfully implemented in Bogotá and Buenos Aires; however, members in Lima preferred to keep their cameras off. In Bogotá, the research team conducted two hybrid (in-person and online) meetings to facilitate group engagement, but they returned to fully online meetings to increase attendance.

Of the 24 local meetings, 20 had over 50% attendance. To encourage attendance, researchers aimed to design the meetings to allow young people to freely share their opinions. For example, facilitators were usually closer to the members’ age. They used visual and dynamic strategies to encourage participation, including presentations with simple language and engaging images, and anonymous live polls and collaborative digital tools like Mentimeter [35]. In Bogotá and Lima, researchers increased meeting frequency to prevent longer gaps between meetings and thus maintain group engagement. However, in Buenos Aires, last minute cancellations by members led to reduced meeting frequency. In those cases, feedback was collected via WhatsApp. For more details on attendance, see Supplementary Material 2.

In February 2023, a Joint LEAP online meeting was held in response to LEAP members’ interest in exchanging insights on regional mental health issues. A total of 16 members attended: 7 from Bogotá, 6 from Lima, and 3 from Buenos Aires.

#### Meetings content and contributions to the study

The first meeting with each LEAP aimed to introduce the research team and LEAP members, as well as give an overview of the study, and the LEAP objectives. Whenever a new member joined the LEAP, the research team scheduled an induction meeting with them. Some of the cross-country topics addressed in the subsequent meetings included brainstorming about recruitment strategies, validating study materials (e.g., clarity, language), asking them to participate in pilot studies, and discussing preliminary study findings and strategies for dissemination of results. Most of the feedback received from the LEAP led to adaptations in study processes and materials wherever feasible and aligned with the study objectives. Table 1 describes the changes made to the OLA Programme based on the LEAP members’ contributions.

**Table 1:** Changes made to the OLA programme based on the LEAPs’ contributions

Study area	Changes made to the OLA Programme based on the LEAP members’ contributions
Study Materials	- Cohort study questionnaire: Changes in wording to facilitate understanding and suggestions on applying questionnaires remotely. - Interview guide for qualitative study: Changes in wording and suggestions on conducting interviews remotely.
Participant Recruitment	- Recruitment spaces: Suggestions of organisations and/or groups that could collaborate with participant recruitment for the various research activities. - Considerations for recruitment: Suggestions to maximise young people’s recruitment and engagement in research activities, especially for a short-term arts intervention (see Supplementary Material 1).
Participation in Pilot Studies	- Experience sampling method pilot study: LEAP members were invited to participate in a pilot of the experience sampling study (see Supplementary Material 1), and gave feedback on app usability and wording in the questionnaire. - Interviews (Bogotá only): LEAP members were invited to pilot and give feedback on the qualitative study interview guide.
Discussion of Preliminary Results	- Interviews (Lima only): The LEAP members contributed to understanding the information the study participants provided during the interviews.
Dissemination of Study Results	It was suggested to consider the following aspects for the dissemination of results: - Definition of target audience(s): Generate content on social media aimed at young people and adults. - Dissemination channels: Importance of using social media in an appealing way. - Content: Study results that may be interesting for different types of target audiences (e.g. according to age).

Additionally, a variety of activities were carried out in specific cities, either led by LEAP members or facilitated by the research teams. For instance, in Bogotá, LEAP members emphasised the necessity of signposting study participants to mental health resources. Although the research teams were already implementing this practice, the LEAP members stressed that the materials should be developed by young people themselves. In response, the Bogotá research team dedicated one meeting for LEAP members to design an infographic, which was subsequently shared on the study’s social media platforms and distributed to study participants in Bogotá. In Lima, the research team proposed a study aimed at evaluating the appropriateness of public mental health services for young individuals. As this study was conceived once the LEAP was already established, members were able to offer their insights on the study’s objectives, design, recruitment strategies, and materials. A detailed summary of each meeting’s agenda, conclusions, and recommendations can be found in Supplementary Material 2.

LEAP members expressed interest in meeting their LEAP peers in the other countries to share experiences of the current mental health situation in their countries and discuss how the study could help address the issues they identified. Each LEAP chose two relevant challenges and two representatives to present their conclusions to the other groups. Members collaboratively developed a series of recommendations highlighting ways in which study findings could bridge the knowledge gap around youth mental health challenges. This feedback was then recorded in video format to be showcased at the annual OLA Programme meeting in March 2023. Following the presentation, the principal investigators from each country responded to the LEAPs, acknowledging the significance of their insights and emphasising how these contributions could enhance the OLA Programme. A summary of the ideas presented in the video can be found in Table 2.

**Table 2:** Messages from the LEAPs to the OLA programme staff

Messages	
1. Psychoeducation	Our society lacks knowledge about youth mental health, and the routes to access care. The OLA Programme could highlight the importance of designing awareness campaigns targeting not only young people with these experiences but also everyone around them, including family and friends.
2. Stigma	Young people with mental health problems fear rejection if they share their feelings. The OLA Programme could provide information on the importance of social support when a loved one reveals a feeling of emotional discomfort.
3. Recognise strategies that do not work	It is important to recognise valuable and useful strategies that improve mood, but it is similarly important to identify ineffective and counterproductive strategies. The OLA Programme explores strategies that help overcome emotional distress, but also those that do not, which could include toxic positivity, avoiding the communication of emotions, or self-harm.
4. Dissemination	Given that young people frequently use social media, the OLA Programme should use strategic channels for disseminating results. In addition, this information must be presented in a visually appealing and concise way.

### Young people’s feedback on the LEAPs

A focus group with 8 members from the three LEAPs was conducted in July 2024 to gather their feedback and overall experience. Table 3 summarises the focus group’s findings.

**Table 3:** Summary of the members’ feedback on the LEAPs

Categories	Codes
Facilitators for LEAP engagement	Personal motivation to participate in the LEAP
	Comfort and connection for mental health discussions with researchers and peers
	Use their lived experience to inform a large-scale project
Challenges	Time constraints due to other responsibilities
	Lack of engagement during the meetings
Recommendations	Communicating with the LEAP more frequently
	Sharing materials used during the meetings
	Holding in-person meetings

### Facilitators for LEAP engagement

LEAP members identified a range of facilitators for their engagement throughout the programme. Most of them mentioned that personal motivations were essential for their decision to participate. The main motivation reported was the desire of helping others by learning new resources to support people around them but also themselves: *“I have friends and family who also go through [emotional distress] […] I like having tools to support them or at least know how to be there for them.” (Female*,* 27*,* Buenos Aires)*. Other reasons include previous interest in mental health-related topics and seeing the programme’s outcomes.

Another facilitator was the comfort and connection for mental health discussions with researchers and peers during the meetings. Researchers were described as “kind”, and members highlighted that they made them feel at ease while discussing different topics and facilitated their interaction with other members during the meetings: *“We get along great. They are always attentive and kind with the group.” (Male*,* 20*,* Lima)*. This allowed them to participate freely in the diverse discussions during the meetings.

Members also reported feeling comfortable with other young people, as they recognised similarities within the group: *“It was really nice to be able to share the space with people who had similar perspectives and experiences as me.” (Female*,* 26*,* Lima)*. One member also described it being easier for him to discuss these topics because he did not personally know the people in the group, which gave him a sense of anonymity: *“You can show yourself as you are.” (Male*,* 19*,* Bogotá)*.

Moreover, some members highlighted the opportunity to use their lived experience to inform a large-scale project. They valued being able to provide insights from their *“own feelings and experiences” (Female*,* 23*,* Bogotá)*. They noted that their feedback and suggestions could be particularly impactful for people with similar characteristics: “*My peers are the ones who also feel this [emotional distress] […] I think [what I share during the meetings] is more important after I personally went through the same.” (Female*,* 27*,* Buenos Aires).* Additionally, they highlighted their age as a relevant factor: *“As a young person with depression*,* I could say ‘Hey*,* this happened to me’ […] I know you are taking that information to help other people my age who are also feeling like this.” (Female*,* 26*,* Lima).* Finally, a small group of members also shared that their feedback played a role in a significant project: *“In some ways*,* I feel like I participated in something truly important.” (Female*,* 27*,* Buenos Aires).*

### Challenges

LEAP members highlighted several challenges they faced in attending and participating in the meetings. Some expressed time constraints related to work, academic commitments, or caregiving responsibilities. For example, two members became mothers during their involvement with the LEAP, leading to new priorities that affected their availability: *“She was a young mom and had to care for her baby.” (Female*,* 23*,* Bogotá)*. Additionally, one member noted that they were providing care for a family member with a health issue, which further impacted their availability to engage consistently.

Regarding the lack of engagement and focus during the meetings, a few members said the online modality made the interaction harder at the beginning: *“It was hard [to participate in the meetings] because we didn’t know each other.” (Female*,* 23*,* Lima)*. The option to keep the microphone or camera off contributed to feelings of disengagement and made it more difficult to establish connections among members. Additionally, another member mentioned occasional connectivity issues, further complicating their ability to fully engage in the discussions.

### Recommendations

A few members recommended that the research teams should communicate with them more often during the programme process to update the group on the study’s progress, especially during periods without meetings: "*Keeping us up to date on the programme’s evolution and progress*,* I think it would be nice to have more information [about it].” (Female*,* 27*,* Buenos Aires).* A smaller group mentioned it would be helpful to share the materials used during the meeting, such as PowerPoint presentations, documents, notes, etc., so that they can revise them when necessary. Finally, some recommended holding in-person meetings to facilitate more fluid communication: *“If we could have in-person meetings*,* we could get to know each other better*,* and I think that would make the communication between all of us better.” (Female*,* 23*,* Lima).*

## Discussion

This paper describes and examines the development and implementation of three LEAPs involving young Latin American people with experiences of emotional distress within the OLA Programme and reports their evaluation of the experience. To our knowledge, these are the first regional LEAPs in Latin America, presenting a unique opportunity for youth engagement in mental health research in the region. The shared language and comparable socioeconomic and cultural backgrounds facilitated meaningful discussions on mental health issues and research priorities. LEAP members contributed to refining data collection tools and materials, giving suggestions to optimise the recruitment and dissemination process, participating in pilot studies and discussing preliminary findings. Although the development of these LEAPs was largely intuitive for the local research teams, due to the lack of similar regional initiatives, and was not exempt from difficulties, the groups remained active throughout the programme’s duration.

While the LEAPs were successfully established, our experience showed some challenges. Initial recruitment strategies, such as outreach through arts organisations in Buenos Aires and Lima, may not have attracted individuals genuinely interested in mental health-related topics. Adjusting the recruitment sites (e.g., universities, mental health centres) might have helped attract youths who were more interested either because they were already receiving emotional support or feeling stress from their studies. Furthermore, research teams will need to anticipate the need of recruiting new members on a rolling basis, as changes in the situation of young people can easily change, leading them to withdraw from the group [36]. In those cases, induction sessions proved to be beneficial for new members to integrate with the group smoothly.

Additionally, socioeconomic barriers may limit participation, as many young people have to balance work and study, making voluntary involvement difficult [37, 38]. Cultural factors might also play a significant role, as mental health is often more stigmatised and young people’s perspectives are less commonly sought and valued in LMICs compared to high-income countries [28]. Even though involving young people in research is becoming more common, it is still not the norm [39]; therefore, they may be unfamiliar with the opportunities to participate in studies from this role. For many youths, it can be difficult to believe that institutions genuinely care about their input because, typically, governments claim that young people’s mental health is a priority, yet they often do not take concrete actions to address this issue [40]. Despite this, shared regional challenges, such as poverty, healthcare access disparities and political instability, allowed young people to engage in meaningful discussions on mental health research at a broader regional level.

To maintain engagement, research teams experimented with different strategies. Increasing meeting frequency and sending updates via WhatsApp helped sustain involvement. However, logistical issues still existed, such as last-minute cancellations in Buenos Aires. As a result, the research teams learned to tailor the frequency of their interactions to each local LEAP to keep them engaged while ensuring their input was truly needed.

Online meetings facilitated attendance by eliminating distance barriers, which can be challenging in large cities like the ones involved in this study. However, they also introduced challenges, such as reduced personal connection, difficulties in reading non-verbal cues, interruptions from family or household responsibilities, and potential technical issues that could hinder engagement [41]. Encouraging camera use helped address some of these barriers, but members in Lima opted to keep them off. While this decision might have helped them feel more comfortable, it may have also led to disengagement. To address some of these challenges, hybrid meetings were introduced in Bogotá to enhance engagement and create a stronger sense of community. However, hybrid formats also present challenges, such as unequal participation dynamics where in-person attendees dominate discussions [42]. Ultimately, the Colombian team decided to revert to remote meetings, as members found it easier to join online, which contradicts the request to do more in-person meetings in the focus group. Research teams must then recognise that there is no predetermined formula for success; instead, they will need to refine their approaches and flexibly adapt to the specific requirements of each LEAP.

LEAP members contributed significantly to some aspects of the research by reviewing study materials, assisting with recruitment strategies, and providing feedback on preliminary findings and dissemination methods, which aligns with existing LEAP models in Europe and North America [2, 43]. Their contributions were significant, particularly in enhancing the relevance and practicality of the research from a young person’s perspective. For instance, the cohort study’s questionnaire was piloted with adolescents and young adults at the start of the study to test its clarity. After some improvements made by the research team, the updated questionnaire was shared with LEAP members for further suggestions and improvements. As a result, most participants of the OLA Programme reported no issues understanding the questions.

Notably, LEAP members also advocated for activities beyond the original study design. Their feedback encouraged the research team to consider supplementary materials aimed at young individuals (e.g., the infographic), facilitated discussions among LEAP peers about mental health challenges (e.g., joint meeting), and provided advice on how to communicate study findings. Moreover, the study addressing mental health policies for young people, which was conceptualised while the LEAP was already established, allowed LEAP members to contribute early and influence key decisions, such as including an exploration of patients’ expectations and experiences of receiving treatment in community mental health centres, which was not initially included in the study.

However, the LEAPs’ involvement in the cohort study occurred only after its core structure was finalised. Establishing LEAPs earlier in the research process might have enabled members to influence study objectives and methodologies more substantially, better aligning the study with our target population’s needs [24]. Nevertheless, establishing LEAPs during the grant application process was not feasible due to structural barriers, including funding constraints and a lack of institutionalized advisory groups in LMICs. These barriers highlight the need for increased financial and infrastructural support to sustain participatory research initiatives beyond individual project timelines to align with more funding bodies requesting the development of LEAPs across studies [44–46].

The LEAP experience was also shaped by power dynamics. While members’ input was highly valued and carefully considered, final decisions rested with the research team, who needed to ensure any changes were within the scope of the grant’s agreements. Furthermore, inherent differences in age, educational background, socioeconomic status and having history of emotional distress may have influenced group interactions [46–49]. The research team tried to address some of these issues by selecting facilitators closer to the members’ age or providing reimbursement for participation in the meetings; however, unintentionally, these may have created other types of issues. For example, principal investigators generally not attending LEAP meetings could have created distance between both actors, and reimbursements could potentially reinforce existing power dynamics [50]. In addition, members were not asked to disclose personal experiences of distress during the meetings, which in practice removes the emphasis on their mental health condition as a conversation start point. While research teams made an effort to create a horizontal structure by promoting an active listening approach, eliminating access barriers to the meetings and showing how feedback was incorporated in the study, this does not guarantee that barriers were completely eradicated, which highlights the complexities of collaborative research.

Nevertheless, LEAP members valued having a safe space to discuss the proposed topics and connect with peers who share similar experiences. These findings align with a study involving young co-researchers with lived experience of mental health problems and previous involvement in research [45]. Members also valued the opportunity to share insights based on their personal experiences, which made them feel like they were contributing to a significant regional project. These aspects may be regarded as benefits identified by the members’ experience of being involved in the LEAPs. Organizations worldwide, such as the Global Mental Health Peer Network (GMHPN) [51], and various regional groups [52], emphasise the positive impact individuals can have on others facing similar mental health challenges.

Moreover, members expressed a strong motivation to join and remain part of the LEAPs, primarily driven by their desire to learn how to support others with equivalent experiences. This is similar to previous studies showing that research involvement is often derived from altruistic motives, such as helping others with similar experiences and using the gained knowledge for good [26, 53]. The GMHPN suggests that participation in such groups not only enhances members’ knowledge about mental health but also promotes empowerment [51].

Finally, when reflecting on the level of involvement of the LEAPs in this study, it is evident that our impact was limited. Based on our experiences and feedback from LEAP members, we believe that providing training is essential. Building basic research skills can empower LEAP members to give better quality feedback on the study [54]. Researchers must also be prepared to learn how their decisions can be effectively informed by LEAPs [28, 55], especially when working with young people [56]. Another recommendation is to give more prominent roles to individuals with lived experience. This could involve exploring additional ways to include them, such as ensuring a LEAP member, instead of research staff, leads the group, or considering a peer researcher as part of the team [57]. As future projects are developed, it is crucial to ensure people with lived experience, particularly young people, gain more space and influence in research studies.

## Conclusions

The experience of implementing the LEAPs in the OLA Programme demonstrates the feasibility of engaging young Latin Americans with lived experience of emotional distress in research. Despite challenges such as recruitment and engagement difficulties, the LEAPs made valuable contributions to study materials, recruitment strategies, and research dissemination. Future initiatives should aim to address structural limitations, enhance participant engagement from initial stages, and establish more equitable collaboration between researchers and young people. This study highlights the importance of integrating youth perspectives in mental health research and the need for institutional support to sustain participatory initiatives in LMICs.

## Electronic supplementary material

Below is the link to the electronic supplementary material.

**Supplementary Material 1**: Description of the OLA programme objectives and activities.

**Supplementary Material 2**: Description of LEAP meetings.

**Supplementary Material 3**

## Data Availability

Data may be available upon request from the corresponding author.

## Abbreviations

LEAP: Lived experience advisory panel
LMIC: Low - and middle-income countries
