# Supplementary material for: Incorporating the voice of young people in mental health research: reflections from three lived experience advisory panels in Latin America
Source: Res Involv Engagem. 2025 Apr 17;11:34. doi: 10.1186/s40900-025-00703-5 (PMC12004765; doi:10.1186/s40900-025-00703-5)
Supplement: Supplementary file 1 — Supplementary Material 1: Description of the OLA programme objectives and activities. [file 40900_2025_703_MOESM1_ESM.docx]

**Supplementary Material 1: Descriptions of the Objectives and Activities of the OLA Programme**
