# Supplementary material for: Incorporating the voice of young people in mental health research: reflections from three lived experience advisory panels in Latin America
Source: Res Involv Engagem. 2025 Apr 17;11:34. doi: 10.1186/s40900-025-00703-5 (PMC12004765; doi:10.1186/s40900-025-00703-5)
Supplement: Supplementary file 2 — Supplementary Material 2: Description of LEAP meetings. [file 40900_2025_703_MOESM2_ESM.docx]

**Supplementary Material 2: Description of LEAP Meetings**

| **Meeting** | **Agenda items** | **Conclusions and Recommendations** | | |
| --- | --- | --- | --- | --- |
|  |  | **Lima** | **Bogotá** | **Buenos Aires** |
| 1  October 2020 | - Introduction of the members, research team and study - LEAP group objectives - Understanding the motivation to join the LEAP - Study recruitment strategies | *Attendance*: *8/8 people*   - Motivation: Having interaction with other young people, contributing to research based on their own experiences. - Recruitment strategies: Get in contact with youth organisations. | *Attendance: 6/8 people*   - Motivation: Desire to have a theoretical approach to the important topics of the study, contribute using life experiences, help other people and participate in a mental health study. | *Attendance: 6/6 people*   - Motivation: The LEAP was enthusiastic about the research proposal and would like to be a part of some of the study activities. |
| 2  February - March 2021 | - Validation of the cohort questionnaire - Discussion on most effective questionnaire application method - Participation in a pilot study with the experience sampling method - Study recruitment strategies | *Attendance*: *3/8 people*   - Questionnaire: Suggestion of editing the language used. - Pilot: Agreement to participate in the pilot. - Recruitment: Recommendations for the design of dissemination materials for the study. | *Attendance: 10/11 people*   - Questionnaire: Slight edits in the wording of the questions: greater depth, terms more commonly used. - Questionnaire application: Due to the context of the pandemic, they deem more appropriate to carry out virtual applications. | *Attendance: 4/6 people*   - Questionnaire: Minor edits to improve comprehension in the Argentinian version. - Pilot: Agreement to participate in the pilot. |
| 3  November 2021 | - Programme’s progress - Sharing future planned activities for the programme in 2022 | A meeting did not take place in Lima due to members’ lack of interest and/or availability to continue with their participation in the LEAP. | *Attendance: 7/11 people*   - Projections for 2022: Suggestions to improve the study implementation with artistic workshops. | A meeting did not take place in Buenos Aires due to members’ lack of interest and/or availability to continue with their participation in the LEAP. |
| 4  July – September 2022 | - Presentation of the new members and study. - Understanding the new members’ motivation to join the LEAP - Programme progress | *Attendance: 6/6 people*   - New members joined the LEAP. - Motivation: Share experiences, help other young people, interest in research. | *Attendance: 8/12 people*   - The programme’s progress was shared and some LEAP members’ questions were resolved. | *Attendance: 6/7 people*   - New members joined the LEAP. - Motivation: Interest in knowing the results of the study. |
| 5*  November – December 2022 | - Programme progress - Preliminary results of the interviews - Artistic workshops study - Collective construction of infographics of mental health self-care actions. - Joint Latin American meeting involving the three LEAPs | *Attendance: 8/9 people*   - Interviews: Comments about the difficulty to understand the questions, agreement with the preliminary results. - Artistic questions: Suggestion to adapt the workshops to the needs and availability or the target audience. The workshop’s theme was deemed as relevant. - Joint Latin American meeting: Interest to know the mental health management in other countries. | *Attendance: 8/12 people*   - Infographic: A joint creative activity is carried out, which brings a greater sense of belonging among the LEAP members. The infographic will be shared with participants with symptoms after the 12-month follow-up. - Joint Latin American meeting: Possible topics to explore in the joint meeting are the response to the programme, statistics, why depression and anxiety appear, OLA Programme progress, etc. | *Attendance: 4/6 people*   - Artistic workshops: Suggestion to adapt the workshops to the needs and availability or the target audience. They emphasise considering the format, the content and the communication channel for the workshops’ recruitment. |
| 6  February 2023 | - OLA Programme progress - Approval of the infographic - Joint Latin American LEAP meeting - Recruitment for the artistic workshops study | *Attendance: 7/9 people*   - Joint Latin American meeting: The following challenges are identified: lack of knowledge about mental health in general, difficulty accepting that one is feeling emotionally distressed, difficulty externalising a mental health problem, fear of rejection for being "different" (having mental health problems) and minimisation of mental health problems. - Artistic workshops: Recommendations for the design of dissemination materials for the study. | *Attendance: 7/12 people*   - Infographic: Approval of the infographic and discussion about the possibility to incorporate it with a mental health programme in the university. - Joint Latin American meeting: The challenges for mental health are selected: access to mental health care, misinformation, prejudices about mental health and lack of validation from other adults | *Attendance: 5/6 people*   - Joint Latin American meeting: The following challenges are identified: Difficulties in accessing health and mental health care, lack of initiative to access treatment, fear of being judged for their mental health problems, overexposure to social networks, excess information, lack of empathy and openness to diversity. |
| 7    April – May 2023 | - Dialogue between LEAP members and principal investigators based on the ideas shared during the Joint Latin American meeting - Reflection on the members’ role in the LEAP | *Attendance: 7/12 people*   - Dialogue between LEAP and researchers: LEAP’s video content reflects members' ideas, and they appreciate that the researcher picks up these ideas. They propose broadcast channels according to the age of the target audience. - Role in the LEAP: They are satisfied by the space, and they propose continuing with joint LEAP meetings. | *Attendance: 6/12 people*   - Dialogue between LEAP and researchers: Discussion on the importance of involving support networks and providing more information about mental health. Finally, the LEAP proposes options to promote education on mental health, such as the dissemination of study results on social networks and the creation of groups of young people trained in psychological first aid. | A meeting did not take place in Buenos Aires due to lack of members’ availability to attend. |
| 8  July 2023 | - Presentation for a sub-study to be conducted only in Lima. - Interview guide review - Recommendations for the interview process | *Attendance: 11/12 people*   - Interview guide: LEAP members advice to use informal language, shorter questions. They also recommended to include other topics: treatment, if interviewees’ expectations are being met. - Interview process: They recommend setting the interview according to the participants availability, showing empathy during the interview, making the process dynamic. | A meeting did not take Bogotá. | A meeting did not take place in Buenos Aires. |
| 9*  November 2023 | - OLA Programme progress - Sharing future planned activities for the programme in 2024 - Case study: Community organisations that promote youth’s emotional wellbeing. | *Attendance: 9/11 people*   - Case study: Selection of organisations that could be included. Recommendations for the inclusion criteria. | *Attendance: 6/12 people*   - The programme’s progress and projections were shared, and some LEAP members’ questions were resolved. - Case study: Recommendations for the inclusion criteria. | A meeting did not take place in Buenos Aires due to lack of members’ availability to attend. |
| 10  June – September 2024 | - OLA Programme progress - Toolkit created by the artistic organisations. | *Attendance: 10/10*   - Toolkit: LEAP members gave suggestions about the content of the document (exercises, used terms), as well as the design (include images to explain the exercises, highlight the titles) | *Attendance: 7/9 people*   - The programme’s progress was shared, and some LEAP members’ questions were resolved. - Toolkit: Given that the document is virtual, the LEAP members gave suggestions about how to navigate the site easily (videos, instructions to access content), as well as strategies to disseminate the information. | *Attendance: 3/7 people*   - Toolkit: LEAP members mainly recommended incorporating images into the document to explain the included exercises. |
| * Meetings conducted in a hybrid format in Bogotá, combining both in-person and online participation. | | | | |
